# A low-glucose eating pattern is associated with improvements in glycemic variability among women at risk for postmenopausal breast cancer: an exploratory analysis

**DOI:** 10.3389/fnut.2024.1301427

**Published:** 2024-04-10

**Authors:** Michelle R. Jospe, Yue Liao, Erin D. Giles, Barry I. Hudson, Joyce M. Slingerland, Susan M. Schembre

**Affiliations:** ^1^Lombardi Comprehensive Cancer Center, Georgetown University, Washington, DC, United States; ^2^Department of Kinesiology at the College of Nursing and Health Innovation, University of Texas at Arlington, Arlington, TX, United States; ^3^School of Kinesiology, University of Michigan, Ann Arbor, MI, United States

**Keywords:** glucose-guided eating, glycemic control, blood glucose self-monitoring, continuous glucose monitoring, food intake regulation

## Abstract

**Background:**

High glycemic variability (GV) is a biomarker of cancer risk, even in the absence of diabetes. The emerging concept of chrononutrition suggests that modifying meal timing can favorably impact metabolic risk factors linked to diet-related chronic disease, including breast cancer. Here, we examined the potential of eating when glucose levels are near personalized fasting thresholds (low-glucose eating, LGE), a novel form of timed-eating, to reduce GV in women without diabetes, who are at risk for postmenopausal breast cancer.

**Methods:**

In this exploratory analysis of our 16-week weight loss randomized controlled trial, we included 17 non-Hispanic, white, postmenopausal women (average age = 60.7 ± 5.8 years, BMI = 34.5 ± 6.1 kg/m^2^, HbA1c = 5.7 ± 0.3%). Participants were those who, as part of the parent study, provided 3–7 days of blinded, continuous glucose monitoring data and image-assisted, timestamped food records at weeks 0 and 16. Pearson’s correlation and multivariate regression were used to assess associations between LGE and GV, controlling for concurrent weight changes.

**Results:**

Increases in LGE were associated with multiple unfavorable measures of GV including reductions in CGM glucose mean, CONGA, LI, J-Index, HBGI, ADDR, and time spent in a severe GV pattern (*r* = −0.81 to −0.49; *p*s < 0.044) and with increases in favorable measures of GV including M-value and LBGI (*r* = 0.59, 0.62; *p*s < 0.013). These associations remained significant after adjusting for weight changes.

**Conclusion:**

Low-glucose eating is associated with improvements in glycemic variability, independent of concurrent weight reductions, suggesting it may be beneficial for GV-related disease prevention. Further research in a larger, more diverse sample with poor metabolic health is warranted.

**Clinical trial registration**: ClinicalTrials.gov, NCT03546972.

## Introduction

Chronic hyperglycemia is an established risk factor for several types of cancer, including postmenopausal breast cancer (1). Evidence shows that breast cancer incidence is increased in patients with diabetes (2) through mechanisms that, in part, involve hyperinsulinemia and insulin resistance (3). While failure to protect glucose homeostasis is pathognomonic of diabetes, people without diabetes also experience hyperglycemia and appreciable glucose variability, particularly among those in early stages of metabolic dysfunction (4–6). Another growing body of evidence indicates that an increased and variable plasma glucose supply, reflected in elevated glycosylated hemoglobin (HbA1c) or elevated glycemic variability (GV), is associated with increased cancer risk, even among people without diabetes (7–10). GV refers to fluctuations in glucose levels or other related parameters of glucose homoeostasis within or between days (11). One pathway whereby GV has been linked to cancer is its induction of oxidative stress through increased postprandial production of reactive oxygen species (12). Previous research has revealed positive associations between oxidative stress and various measures of GV among people with type 2 diabetes (13–16), although this relationship has not always been consistently observed (17). Oxidative stress is a known cause of insulin resistance (12) and increased breast cancer risk (18, 19). In conventional, therapeutic intervention settings, improved GV has been associated with reduced oxidative stress among people with diabetes (16). While anti-diabetic medications are the predominant first line of treatment for diabetes management, these treatments are not usually appropriate for or well tolerated by people without diabetes. Rather, behavioral interventions would be the expected approach for improving glycemic variability in non-diabetic populations. Yet, there remains a paucity of clinical trials targeting GV with dietary modifications, and the findings are inconsistent (20–23). More research is needed to determine if (and what type of) dietary interventions reduce the risk of cancer by favorably modulating GV in populations at elevated metabolic risk without diabetes.

Despite its potential as a modifiable biomarker of chronic disease risk, there have been few behavioral interventions that specifically target GV. Most behavioral interventions target weight reductions. An exemplary behavioral weight reduction intervention is the Diabetes Prevention Program (DPP). The DPP is regarded as one of the most effective and highly disseminated weight loss interventions. However, it is among the most costly and intensive interventions of its kind (24). Recent advances in wearable technology now enable the continuous monitoring of glucose and GV in real-time, offering a unique opportunity to implement glucose-based biological feedback as part of precision health interventions. Low-touch, continuous glucose monitoring (CGM)-based interventions that more directly target glucose-related outcomes, particularly those delivered on digital health platforms (smartphones), are potentially better poised for application to the general population. Previously published research from us and others have indicated the acceptability of CGM in these non-traditional populations (25–28). Further studies are needed to identify how CGM can best be used in interventions targeting chronic disease prevention.

One such CGM-based disease prevention intervention with great potential for scalable implementation is Glucose-Guided Eating (GGE), formerly known as Hunger Training (29–31). In contrast to comprehensive lifestyle interventions, GGE implements a low-contact, timed eating approach, previously shown to be practical for delivery in clinical care settings (32). Rooted in the principles of chrononutrition, which emphasizes the role of meal timing in disease risk (33), GGE offers a unique approach to timed-eating. GGE uses glucose as a personalized biomarker of short-term energy availability, helping individuals differentiate between a desire to eat (hedonic hunger) and a genuine need to eat (non-hedonic hunger). By doing so, it guides personalized decisions about meal timing, ensuring alignment with one’s ability to metabolize carbohydrates (29–31). Like other timed-eating strategies, such as time-restricted eating (33), GGE does not impose specific dietary restrictions. Instead, its primary objective is to optimize energy intake times, ideally when glucose levels are beneath a personalized threshold, akin to morning fasting glucose levels (34). This nuanced approach integrates both the chronobiological insights of when to eat with the metabolic cues of what the body needs. Over a 2–4 week period, participants are trained to eat when two conditions are met: (1) the desire to eat is present and (2) current, preprandial glucose levels are at or below their personalized threshold. This pattern of eating, when glucose is low (“low-glucose eating,” LGE), has resulted in clinically relevant weight losses, increased insulin sensitivity and reduced HbA1c in populations without diabetes (30, 35), and improved metabolic and cancer risk biomarkers among women with obesity who are at risk for postmenopausal breast cancer of a magnitude similar to that produced by time-restricted eating in similar populations (26). To date, however, the promise of LGE to modify measures of GV has yet to be investigated as a possible cancer prevention strategy.

Here, we conducted a secondary data analysis of a 16-week randomized controlled trial (RCT) that examined the feasibility and preliminary efficacy of adding Glucose-Guided Eating to the Diabetes Prevention Program (DPP) in obese postmenopausal women without diabetes who were at high risk of breast cancer. CGM based measures of GV were assessed as exploratory outcomes of the parent study. The goal of this study was to examine associations between changes in low-glucose eating and concurrent changes in GV. Based on prior analyses (26), we hypothesized that greater adoption of low-glucose eating would result in greater improvements in measures of GV. This would be desirable since high GV has been previously linked to increased cancer risk.

## Materials and methods

This was a secondary, observational analysis from the Take Charge trial (31), a 16-week RCT that examined the feasibility and preliminary efficacy of adding a GGE to the Diabetes Prevention Program (DPP) in *N* = 50 predominantly obese postmenopausal women at high risk of breast cancer without diabetes. The DPP is a 2-h weekly group program that covers healthy eating, physical activity, stress management, and behavior change to help prevent or delay the onset of diabetes in those at high risk. The GGE protocol was adapted from prior research (35, 36) and consisted of up to 3 weeks of unblinded CGM-assisted LGE training during which the women learned to eat based on symptoms of hunger they experienced with their glucose levels neared fasting (averaged from two, morning fasting glucose levels). Women were randomized (1:1) to a DPP-only group or a DPP + GGE group. LGE training began at study week 3 for those in the DPP + GGE group. Following the training period and for the remainder of the trial, LGE was encouraged without the assistance of CGM and was assessed pre- and 8-weeks post-intervention. The study protocols were approved by The University of Texas MD Anderson Cancer Center Institutional Review Board and registered with ClinicalTrials.gov (NCT03546972). A full description of the study procedures and primary results have been published elsewhere (31).

Primary findings of the parent GGE study, which were related to feasibility (study accrual, retention, and LGE adherence rates), showed that adding GGE to the DPP was feasible. Secondary effectiveness outcomes did not suggest a synergistic effect of GGE and the DPP on changes in body weight or the cancer-related serum biomarkers assessed in the parent study (31). In subsequent, *ad hoc* analysis, we found that improvements in LGE were variable and experienced by women in both the DPP-only and DPP + GGE arms; a phenomenon that has yet to be explained. Collectively, these findings provide support for merging data from the DPP-only and DPP + GGE arms for the current secondary analysis of the associations between changes in low-glucose eating and measures of GV. Additional details on the methods related to the current study are provided in the sections below.

### Participants

For the original RCT, a cohort of 50 postmenopausal women with a body mass index (BMI) greater than 27 kg/m^2^ were enrolled in the study between 2016 and 2018. Eligible women were postmenopausal and had a high risk of developing breast cancer defined as a Gail model lifetime risk score greater than 20% or a 5-year risk score greater than 1.66%, a history of deleterious BRCA1/2 mutation or Mantle radiation, a history of ductal cancer *in situ*, or a history of high-risk premalignant breast lesions. Women were excluded if they reported being treated for cancer other than non-melanoma skin cancer, were unwilling to use CGM, had a diagnosis of type 1 or type 2 diabetes, were using oral antidiabetic agents (except metformin), were currently undergoing any insulin regimen or GLP-1 receptor agonist treatment, had a measured fasting blood glucose level exceeding 126 mg/dL or HbA1c level exceeding 6.4%, were not proficient in the English language, or did not have daily internet access or an ability to take digital time-stamped photographs.

In the current study, women from the parent project were included if they had at least 3 valid days of plausible, blinded CGM data and time-stamped dietary intake at weeks 0 and 16. A valid day was defined as having at least two time-stamped eating events with plausible, corresponding CGM data. CGM data were considered plausible if a majority of the wear time (>95%) was spent within or above the physiological range for this non-insulin treated population of women without diabetes (≥70 mg/dL). In most instances, women were excluded from the current analysis for having fewer than 3 days with at least two reliable time stamped eating events at either the week 0 or week 16 time points. Only one participant’s week 16 CGM data were deemed implausible due to aforementioned reasons as well as a lack of consistency with their week 0 CGM data. After removing this participant’s data from the analysis, the analytical dataset consisted of 17 participants.

### Measures

#### Low-glucose eating

Low-glucose eating was defined at the level of the individual as the percent of reported eating events with preprandial glucose levels at or below personalized glucose thresholds (31). CGM data were obtained by blinded CGM (FreeStyle Libre Pro, Abbott Diabetes Care, Inc.) worn at weeks 0, 8, and 16 for up to 10 days at a time. The timing and dietary composition of eating events were obtained by image-assisted food records collected using MyFitnessPal. Images of consumed meals and snacks (collectively referred to as meals from here) were captured by the study participants using their personal smartphones and emailed to the study staff. Time-stamps from the smartphone images were recorded in duplicate by independent, trained staff. Reported mealtimes were confirmed by the study dietitian using the time-stamped food photos that were matched to MyFitnessPal records with noted mealtimes. Dietary intake (energy and macronutrient composition) was estimated by a trained research dietitian who transferred the digital diet records into the University of Minnesota Nutrition Data System for Research software (NDSR). Discrete and valid eating events were defined as energy intake from foods or beverages ≥25 kcals that occurred at least 15 min apart. In the event that ≥2 meals were reported as being consumed within 15 min, the dietary composition of the individual events was aggregated and assigned the earliest chronological timestamp. To quantify the number of valid eating events meeting the definition of LGE, food records with mealtimes were merged with the CGM data within 5 min of the time-stamped meals. Eating events that occurred when glucose levels were at or below personalized thresholds were identified as LGE events. LGE was derived as the percentage of total included eating events.

#### Glycemic variability

Glycemic variability (GV) was reported in two ways. First, measures of GV were calculated using EasyGV (a software that calculates GV based on CGM data) (37) and included average daily risk ratio (ADDR), continuous overlapping net glycemic action (CONGA), Glycemic Risk Assessment of Diabetes Equation (GRADE), High Blood Glucose Index (HBGI), Low Blood Glucose Index (LBGI), Lability Index (LI), mean amplitude of glucose excursions (MAGE), and mean of daily differences (MODD) (38). CONGA and LI intervals were set at 60 min, and the *M*-value reference was set at 120 mg/dL. Our sample population had slightly greater GV than a reference population of men and women without diabetes (38). Second, the fraction of time spent in a low, moderate, and severe GV pattern was calculated using a glucotyping calculation tool (39).^1^ This method of glucotyping is based on spectral clustering, which was used to classify different patterns of glycemic responses based on their variability. The fraction of time spent in the different variability patterns correlates with standard measures of glycemia associated with diabetes risk.

### Statistical analysis

Using the derived analytical dataset, associations between changes in LGE and measures of GV were computed as Pearson’s product moment correlation coefficients. Multivariate regression models were used to examine the independent associations between LGE and measures of GV after controlling for concurrent changes in body weight. Statistical analysis was performed using R (version 4.2.0).

## Results

Seventeen women were included in our analytical dataset. The women were predominantly middle-aged, white, partnered, college-educated, with a BMI in the obese category (Table 1). Ten participants (58.8%) had HbA1c (*n* = 8) or fasting glucose (*n* = 8) in the prediabetes range. The remaining women were metabolically healthy (HbA1c <5.7%).

**Table 1:** Participant baseline characteristics.

Variable	*n* (%)^a^
*n*	17
Partnered	16 (94%)
College education or greater	15 (88%)
Employed	11 (65%)
Not Hispanic or Latino	17 (100%)
White	17 (100%)
Age, years, mean (SD)	60.7 (5.8)
BMI 27.5–29.9 kg/m^2^	3 (17.6%)
BMI 30–34.9 kg/m^2^	9 (52.9%)
BMI > 35 kg/m^2^	5 (29.4%)
HbA1c < 5.7%	8 (47.1%)
HbA1c 5.7–6.4%	8 (47.1%)
HbA1c not measured	1 (5.9%)
Fasting glucose <99 mg/dL	8 (47.1%)
Fasting glucose 100–125 mg/dL	8 (47.1%)
Fasting glucose not measured	1 (5.9%)

^a^Unless otherwise specified.

Sixteen-week changes in LGE, body weight, and GV were variable (Table 2). In particular, there was a wide range of changes in LGE. One quarter of participants reduced their LGE by 27–60 percentage points (pp); another quarter reduced it by 0–27 pp; a quarter increased it by 0–24 pp; and the last quarter increased it by 24–62 pp. Change in LGE was not associated with change in body weight (*p* = 0.575) or study arm assignment (*p* = 0.828).

**Table 2:** Baseline, post-intervention, and changes in low-glucose eating, body weight, and glycemic variability.

	*n*	Week 0 mean (SD)	*n*	Week 16 mean (SD)	Change mean (95% CI)
Low-glucose eating (%)	17	29.6 (21.4)	17	30.9 (29.4)	1.3 (−19, 21.5)
Weight (kg)	17	92.5 (18.5)	17	84.7 (19.1)	−6.9 (−9.0, −4.8)
CGM glucose mean (mg/dL)	17	96.9 (8.1)	17	100.8 (9.3)	3.2 (−2.6, 9.0)
CGM glucose standard deviation (mg/dL)	17	0.9 (0.1)	17	1.0 (0.3)	0.1 (−0.1, 0.2)
CONGA	17	4.8 (0.4)	17	5.0 (0.5)	0.2 (−0.1, 0.5)
MAGE (mmol/L)	17	2.1 (0.7)	17	2.5 (0.8)	2.5 (−3.1, 8.2)
J-Index	17	12.9 (2.0)	17	14.0 (3.1)	1.1 (−0.5, 2.6)
ADDR	17	2.7 (1.7)	17	3.5 (3.1)	0.8 (−0.8, 2.4)
HBGI	17	0.9 (0.5)	17	0.9 (0.7)	0.1 (−0.3, 0.5)
LI	17	1.1 (0.4)	17	1.1 (0.6)	0.1 (−0.1, 0.3)
MODD	17	0.8 (0.2)	17	0.9 (0.2)	0.1 (0, 0.2)
GRADE	17	0.4 (0.2)	17	0.5 (0.2)	0.0 (−0.1, 0.2)
M-value	17	3.0 (2.4)	17	2.5 (1.7)	−0.5 (−2, 1.1)
LBGI	17	2.3 (1.4)	17	2.0 (1.1)	−0.3 (−1.2, 0.6)

ADRR, Average daily risk ratio; CONGA, Continuous overlapping net glycemic action; GRADE, Glycemic risk assessment of diabetes equation; HBGI, High blood glucose index; LBGI, Low blood glucose index; LI, Lability index; MAGE, Mean amplitude of glucose excursions; and MODD, Mean of daily differences.

**Figure 1:**
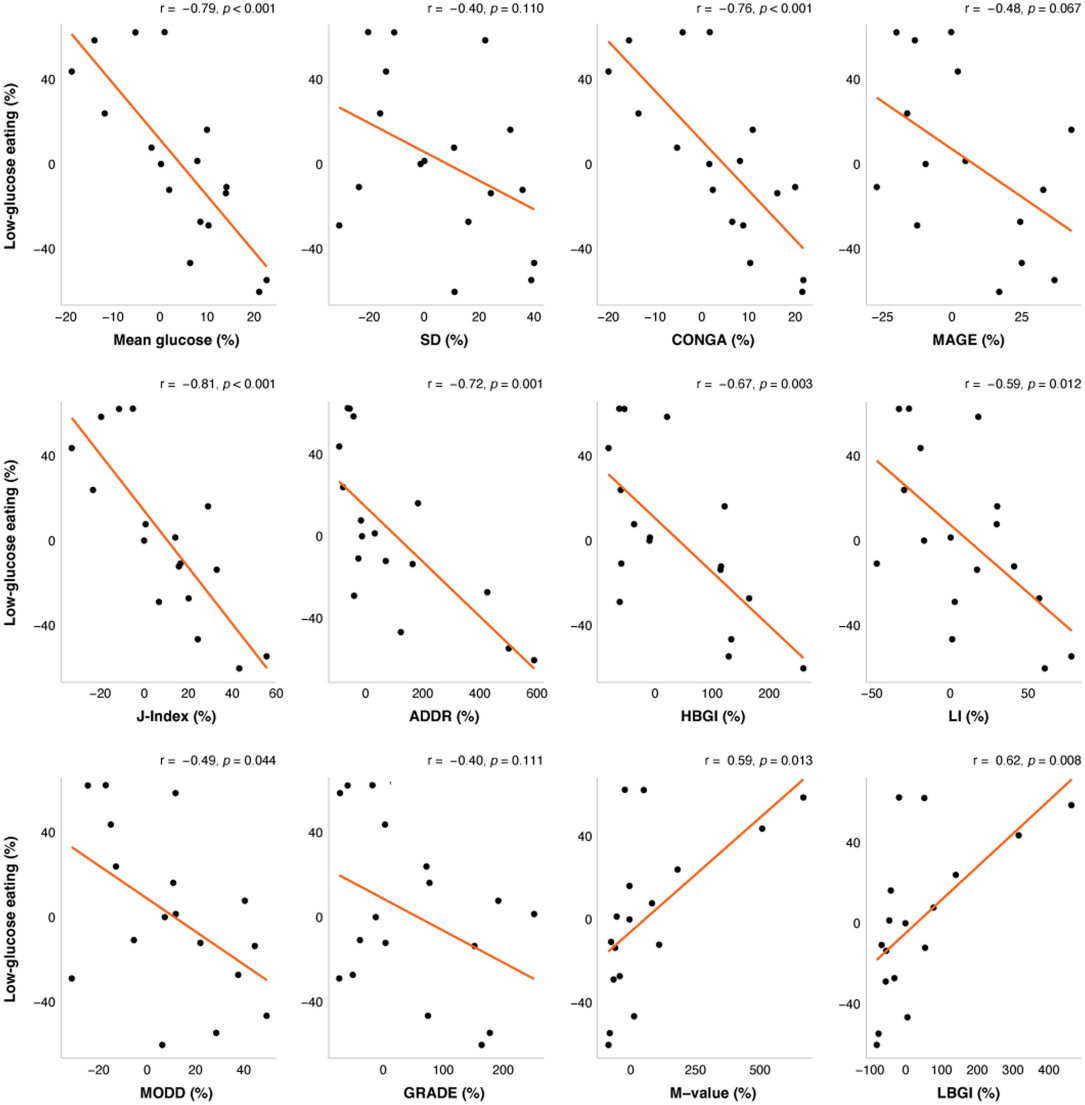
The correlations between change in low-glucose eating (%) at week 16 and changes in glycemic variability (%). The orange line represents the fitted linear model and each point represents one participant. *r* = Pearson’s correlation coefficients. ADRR, Average daily risk ratio; CONGA, Continuous overlapping net glycemic action; GRADE, Glycemic risk assessment of diabetes equation; HBGI, High blood glucose index; LBGI, Low blood glucose index; LI, Lability index; MAGE, Mean amplitude of glucose excursions; and MODD, Mean of daily differences.

**Figure 2:**
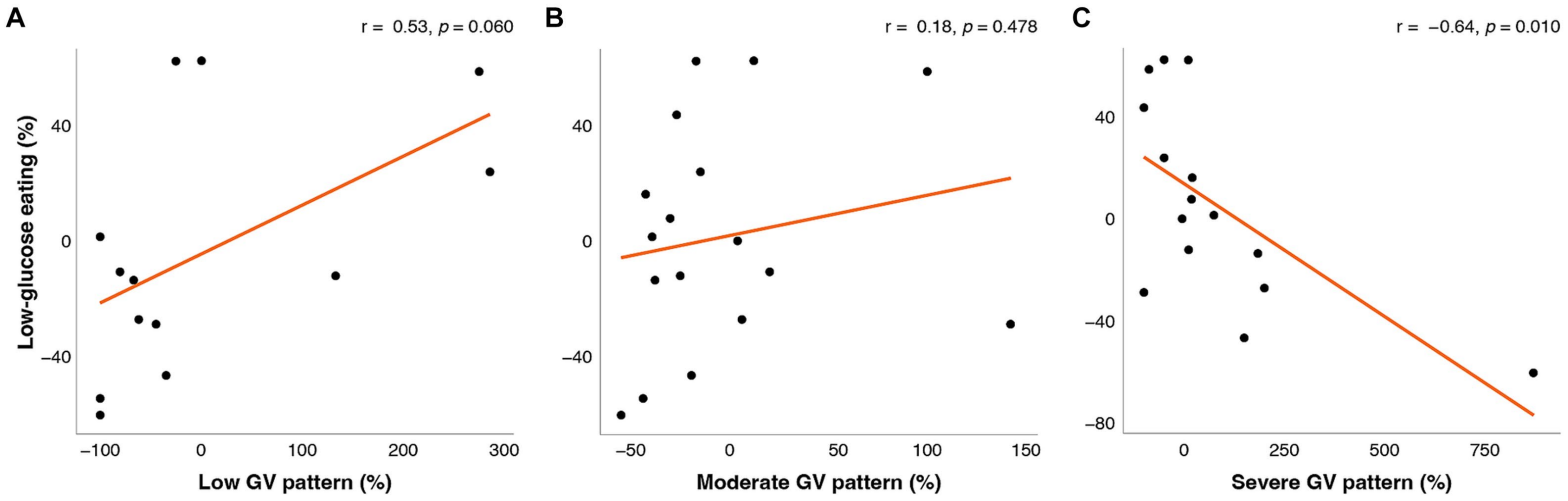
Correlations between change in low-glucose eating (%) at week 16 and change (%) in the fraction of time spent in **(A)** low GV pattern, **(B)** moderate GV pattern, and **(C)** severe GV pattern. The orange line represents the fitted linear model and each point represents one participant. *r* = Pearson’s correlation coefficients.

The change in LGE from week 0 to week 16 was significantly correlated with changes in mean glucose from CGM, CONGA, LI, J-Index, ADDR, HGBI, LBGI, and M-value (Figure 1), and time in a severe GV pattern (Figure 2). Weight changes were independently associated with changes in CGM glucose mean, CONGA, LBGI, and M-value in the multivariate regression models (*p*s < 0.02). After controlling for weight changes in multivariate regression models, only the change in MODD was no longer associated with changes in LGE (*p* = 0.113). The resulting regression estimates indicated that for every 10 percentage point increase in LGE, there was a 2% decrease in mean glucose (*p* < 0.0001), 2% decrease in CONGA (*p* = 0.002), 5% decrease in J-index (*p* < 0.0001), and 50% decrease in severe GV pattern (*p* = 0.018). Changes in LGE and weight explained 33–67% of the change in these GV measures (adjusted *R*^2^).

## Discussion

This secondary data analysis of a 16-week dietary intervention RCT examined associations between changes in LGE and measures of GV in predominantly obese postmenopausal women without diabetes who were at high risk of breast cancer. Changes in LGE explained a significant amount of the variance in changes in several measures of GV (25–60%) and these remained significant after accounting for weight changes. Despite strong effect sizes, the magnitudes of the observed effects were relatively small and might not be generalizable to more diverse populations who might be at greater metabolic risk of breast cancer. Nevertheless, modest changes in LGE were observed to favorably modulate GV to an extent that is comparable to prior dietary and therapeutic interventions. As such, this study provides support for future, more definitive examination of the efficacy of LGE on GV and other related markers of cancer risk (e.g., oxidative stress, insulin resistance) as a chronic disease prevention strategy for women at risk for postmenopausal breast cancer with suboptimal metabolic profiles.

Previous studies that examined the association of meal-timing patterns with GV have primarily focused on time-restricted eating (40–43). The results to date have shown a benefit of time-restricted eating on GV, but only among adults with less favorable metabolic profiles (42). There does not appear to be an effect of time-restricted eating on GV in adults without diabetes (40, 41). Other dietary interventions, including low carbohydrate, low glycemic index, and low glycemic load meals or diets, have been shown to reduce GV among adults with and without diabetes (20, 22, 23, 44). In these studies, significant reductions in MAGE, the most common measure of GV, have been observed (−0.3 to −0.8 mmol/L) (20, 23, 44); however, effects of this magnitude do not appear to modify markers of pancreatic β-islet cell function or oxidative stress in adults without diabetes (45). In the current study, where nearly half of the women were metabolically healthy and the remaining half had prediabetes, we observed magnitudes of effect that were comparable to prior intervention studies in adults with similar metabolic profiles (46). It is most likely that these significant but modest effects can be explained as floor effects. As such, it remains plausible that dietary interventions with favorable effects on GV, including GGE/LGE, could be effective GV-related disease prevention strategies, particularly among those at greater metabolic risk.

There are notable strengths and limitations of this current study. One strength was the use of up to 10 days of blinded CGM data to estimate GV at each assessment point. Comparably, a majority of studies use a maximum of 3 days of CGM data at each assessment point. It would be expected that more CGM data would be less sensitive to days with less characteristic GV profiles and result in a better reflection of usual GV. Additionally, our use of blinded CGM data ensures that the observed effects are unrelated to any potential confounding effects of viewing glucose trends in real-time. We also observed a substantial amount of variability in changes in LGE and GV, despite being in the context of an intervention, which enabled us to conduct these analyses. Despite these strengths, the study included a relatively small and demographically homogeneous sample of predominantly obese, but otherwise metabolically healthy women at risk for postmenopausal breast cancer. While future research examining the effect of LGE on GV will need to be more diverse and target women who are at greater metabolic risk, doing so has the potential to strengthen both the generalizability and clinical relevance of future findings. Another potential limitation was defining LGE as the percentage of total included eating events. However, using a relative vs. absolute definition of LGE allowed for comparison across study participants and addressed, in part, limitations arising from missing meals that were either not reported by study participants or did not have a confirmed timestamp, which was <5% of total days. Lastly, the exploratory nature of the analysis does not allow us to draw conclusions about the causality of the observed associations or the potential of GV to mediate previously observed effects of LGE on markers of cancer risk (e.g., insulin resistance) (26) or oxidative stress. That said, the results of the current study can be used to inform the design and sample size estimation for a future study with these aims.

## Conclusion

In summary, this study confirmed our hypothesis that greater adoption of LGE would result in greater improvements in measures of GV. However, it remains unclear whether LGE can induce a magnitude of change in measures of GV that are clinically meaningful or result in a protective effect on biomarkers of cancer risk. Nonetheless, the results of this exploratory, secondary analysis provide justification for a larger, efficacy RCT that tests this hypothesis in a more diverse population of women at risk for postmenopausal breast cancer where GV is formally tested as an underlying mechanism of risk reduction.

## Data availability statement

The datasets presented in this study can be found in online repositories. The names of the repository/repositories and accession number(s) can be found at: Zenodo at https://doi.org/10.5281/zenodo.7992581.

## Ethics statement

The studies involving humans were approved by the Institutional Review Board of the University of Texas MD Anderson Cancer Center (protocol 2017-0507). The studies were conducted in accordance with the local legislation and institutional requirements. The participants provided their written informed consent to participate in this study.

## Author contributions

MJ: Formal analysis, Visualization, Writing – original draft, Writing – review & editing. YL: Writing – review & editing. EG: Writing – review & editing. BH: Writing – review & editing. JS: Writing – review & editing. SS: Writing – original draft, Conceptualization, Formal analysis, Funding acquisition, Project administration, Writing – review & editing.

## Footnotes

^1^ https://adaychen.shinyapps.io/shinyspecclust/
